# Sex-Specific Movement Responses of Reeves’s Pheasant to Human Disturbance: Importance of Body Characteristics and Reproductive Behavior

**DOI:** 10.3390/ani12131619

**Published:** 2022-06-23

**Authors:** Shuai Lu, Zhengxiao Liu, Shan Tian, Kai Song, Qian Hu, Jianqiang Li, Jiliang Xu

**Affiliations:** 1 School of Ecology and Nature Conservation, Beijing Forestry University, Beijing 100083, China; ls0508@126.com (S.L.); liuzhengxiao@bjfu.edu.cn (Z.L.); doriatshan@163.com (S.T.); huqian1995@bjfu.edu.cn (Q.H.); lijianqiang@bjfu.edu.cn (J.L.); 2 Key Laboratory of Animal Ecology and Conservation Biology, Institute of Zoology, Chinese Academy of Sciences, Beijing 100101, China; songkai2014@sina.com

**Keywords:** Galliformes, human-modified habitat, human presence, reproductive behaviors, satellite tracking

## Abstract

**Simple Summary:**

Human disturbance has a strong impact on the movement of wild animals. The Reeves’s Pheasant is listed as an endangered species by the International Union for Conservation of Nature (IUCN) and a nationally protected species in China. This study evaluated how the movement patterns of this species responded to human disturbance. We observed large differences in movement characteristics between sexes during the breeding season of Reeves’s Pheasants, and found that reproduction had a significant effect on the movement of females. Males shifted their movement peaks to earlier times in the day to avoid the presence peaks of humans. The greater the distance to human-modified habitat, the higher the movement intensity of males, and the lower the movement intensity of females. This study suggested that the potential impacts of different forms of human disturbance on wildlife should be considered in future conservation planning.

**Abstract:**

Human disturbance has a strong impact on the movement of wild animals. However, it remains unclear how the movement patterns of the Reeves’s Pheasant (*Syrmaticus reevesii*) respond to human disturbance in human-dominated landscapes. We tracked the movement of 40 adult individual Reeves’s Pheasants during the breeding season, and used the dynamic Brownian bridge motion model and kernel density estimation to analyze the diurnal movement patterns of Reeves’s Pheasants and their response to human presence. We analyzed the paths of Reeves’s Pheasants based on a partial least squares path model, considering habitat conditions, body characteristics, and reproductive behaviors. We found that males had two clear diurnal movement peaks, whereas reproductive and non-reproductive females did not show such movement peaks. Males shifted their movement peaks to earlier times in the day to avoid the presence peaks of humans. The correlation between human-modified habitat and the movement intensity of Reeves’s Pheasant differed between sexes. For males, the distance to forest paths had a positive correlation with their movement intensity through affecting body conditions. For females, the distance to forest paths and farmland had a negative correlation with their movement intensity through affecting habitat conditions and reproductive behaviors. Our study provides a scientific basis for the protection of the Reeves’s Pheasant and other related terrestrial forest-dwelling birds.

## 1. Introduction

Animal movement is a fundamental force shaping population dynamics, population interactions, and the environment [1]. As human activities expand in natural areas, human disturbance, such as human presence and human-modified habitats [2], has a strong impact on the movement of wild animals [3]. Human presence can make wild animals experience intense fear [4], so animals may adjust their movement patterns to avoid contact with human beings [2]. Grazing [5] and companion animals such as dogs and cats [6,7] also can affect wildlife movement. As with natural predator–prey systems, such risks have nonlethal effects on the physiology and fitness of wildlife [8,9]. At present, at least 95% of the terrestrial land area (excluding Antarctica) has been altered by anthropogenic disturbance [10], and alterations of natural habitats have exerted a profound influence on wildlife movement [3]. However, few studies have considered the simultaneous effects of different types of human disturbance on the movement of wildlife [2]. In-depth studies on the impact of different types of human disturbance on wildlife movement are needed as they can shed new insight into space management and conservation planning [2,11] and make contributions to wildlife protection [12,13].

Human disturbance influences wildlife movement in both direct and indirect ways. For example, human disturbance can directly control the movement patterns of wild animals [3], and indirectly regulate wildlife movement through affecting body size [14,15]. However, such indirect effects are largely ignored in current studies. So far, most studies have only confirmed that human disturbance has a significant impact on wildlife movement [3], but have not pointed out how human disturbance affects it. In addition, previous studies on wildlife movement behaviors have generally focused on mammals [2,3,16,17,18], and fewer studies have paid attention to birds such as galliforms [19]. As a component of the ecosystem, galliforms play an important role in maintaining ecosystem stability, and can be regarded as an effective indicator of local forest quality and conservation status [20]. Many wild galliforms are endangered because of their high environmental sensitivity and low reproductive rate [21]. Under the rapid expansion of human activities in natural areas, a better understanding of the population status and response to human disturbance of galliforms is particularly important for the protection of galliforms species.

The Reeves’s Pheasant (*Syrmaticus reevesii*) is a galliforms species endemic in China [22]. It has been listed as vulnerable in the International Union for Conservation of Nature Red List [23]. As typical terrestrial forest-dwelling birds, Reeves’s Pheasant females nest alone on the ground and are particularly sensitive to habitat changes [21]. As a nationally protected species in China, it is a flagship species in some mountain regions in central China, where it was once widely distributed [24]. The Reeves’s Pheasant is sexually dimorphic and the males have colorful feathers and a quite long tail. Its tail feathers are used as props in Chinese opera, and thus this species possesses high cultural and economic value. Illegal hunting driven by interests was a major threatening factor for the Reeves’s Pheasant in the 1990s [21]. Poison baits used by farmers to protect their crops have caused additional mortality of this species [25]. Data from field surveys showed that the distribution and populations of the Reeves’s Pheasant have decreased dramatically since the 1970s [25]. Because of the sharp decline in population, the Reeves’s Pheasant was listed in Appendix Ⅱ of the Convention on International Trade in Endangered Species of Wild Fauna and Flora [26]. After long-term environmental modification by human activities (such as agricultural land, residential areas, paved roads, and tourism facilities) and their continuous expansion, human disturbance has become one of the most important factors affecting the survival of most species including the Reeves’s Pheasant [21,27,28].

Reeves’s Pheasants are highly vigilant [21]. Despite substantive challenges to locating and observing this animal, detailed behavioral observations based on radiotelemetry and infrared camera technology have illuminated the basic movement ecology of this species. The Reeves’s Pheasant is a resident bird with poor dispersal ability and strong fidelity to home range [21]. Its dispersal model remains unclear. During the breeding season, male Reeves’s Pheasants occupy territories (from March to June), and female Reeves’s Pheasants visit multiple male territories to choose mates [21]. The Reeves’s Pheasant is a diurnal species and most of their activities occur during 5:00–19:00 [29]. Reeves’s Pheasants have two daily movement peaks, males at 7:00–9:00 am and 17:00 pm, females at 9:00–11:00 am and 17:00 pm [30]. During the incubation period, reproductive females remain in the nest almost all day [21]. Few studies, however, have explored the movement characteristics of Reeves’s Pheasant in response to human disturbance [29].

The breeding season is a vital life-cycle stage for wildlife. In this study, we aimed to (1) understand whether there are differences in movement characteristics between sexes during the breeding season of Reeves’s Pheasants, and whether female reproductive behavior causes changes in their movement patterns; (2) estimate the response of the diurnal movement patterns of Reeves’s Pheasants to the aggregate presence of people and livestock, and explore whether the presence of people and livestock poses the same threat to the Reeves’s Pheasant; and (3) evaluate the effects of human-modified habitats on the movement intensity of Reeves’s Pheasants, and the patterns and the differences between sexes.

## 2. Materials and Methods

### 2.1. Study Area and Data Collection

The study site was located in Pingjingguan village, Guangshui City, Hubei Province, China (113°54′09″–113°55′21″ E, 31°51′03″–31°52′40″ N). It is one of the main distribution areas of the Reeves’s Pheasant [31]. The land use types include coniferous forest, deciduous broad-leaved forest, coniferous broad-leaved mixed forest, undergrowth, wasteland, water sources, and human-modified habitat, such as farmland, forest paths, and residential and paved roads.

We used satellite trackers (designed and produced by Druid Technology Co., Ltd., Chengdu, China) to monitor the movement of 40 adult individuals (24 females and 16 males) during the breeding season (From March to June, sunrise: 5:20–6:49, sunset: 18:22–19:30) in 2020 (10 females and 9 males) and 2021 (14 females and 7 males). The tracker weighed about 20 g, which was less than 3% of the body weight of an adult Reeves’s Pheasant individual. The frequency of data recorded by the tracker was once per hour. We measured the physical characteristics of the birds (wingspan, tail feather length, body weight, body length, and tarsus length) after they were caught for attaching satellite trackers.

To investigate the presence and rhythms of people and livestock (dogs, cats, cattle, and sheep), 69 infrared cameras (Ltlacorn-6310 wmc model produced by Zhuhai Lieke Electronics Co., Ltd., Zhuhai, China) continuously monitored the area from March to June 2021, and the shooting mode was set as taking two photos and a 15 s video once triggered with trigger interval of 30 s [30]. The camera was fixed on a small or midsize tree or shrub at 20–50 cm above the ground. Cameras were put at intervals of more than 100 m.

### 2.2. Movement Characteristics of Reeves’s Pheasants during the Breeding Season

We categorized female individuals into reproductive (*n* = 18) and non-reproductive (*n* = 6) groups according to whether the female was incubating, and abandoned the data related to the incubation period of reproductive females (based on the tracking data). The final data set was composed of 41,047 positioning locations that were selected for analysis (Table A1).

Dynamic Brownian bridge movement models (dBBMMs) are often used together with satellite tracking technology, because the dBBMM considers both time autocorrelation and large data sets and can process irregular sampling data [32]. We used the package “move” [33] to calculate Brownian motion variances ($\sigma_{m}^{2}$) for each time step (i.e., the spatiotemporal changes between consecutive positioning locations) and estimated the 95% utilization distribution (95%UD) of each individual using the dBBMM in R 4.0.3 [34]. $\sigma_{m}^{2}$ provides a measure of an animal’s movements: the lower the value, the lower the intensity of movement [35]. 95%UD represents the home range [35]. The dBBMM considered the elapsed time between consecutive locations (temporal autocorrelation) as well as the location error. We evaluated the position accuracy of the tracker according to the location data of the nest site, and the location error of all locations was estimated to be 10 m. dBBMMs estimate $\sigma_{m}^{2}$ by incorporating behavioral change-point analysis [32]. To determine if a behavioral change occurred along a trajectory, the dBBMM relies on a user-defined sliding window encompassing *n* locations along the movement trajectory. Then, behavioral change-points are identified by comparing movements within a specified window of consecutive locations to a specified margin of prior locations directly preceding the window [35]. The choices of the window and margin sizes should match the time interval within which behavioral changes are expected to occur [35]. Following this recommendation and after visual inspection of our data, we chose a window of seven locations (corresponding to approximately 7 h) with a margin of three locations.

We calculated the mean $\sigma_{m}^{2}$ (5:00–19:00) of each individual as the individual’s movement intensity during the breeding season, and examined whether there are significant differences in movement intensity among males, non-reproductive females, and reproductive females. The mean individual movement intensity was normally distributed through natural logarithm transform (Kolmogorov–Smirnov Z test) and a linear mixed model (LMM) was used with the mean individual movement intensity as dependent variable and categories as independent variables. This part of the analysis was carried out using SPSS 22.0.

### 2.3. Response of the Movement Patterns of Reeves’s Pheasants to Human Presence

We defined counts over 24 h as valid camera days for each camera [36]. Photos and videos of individuals of the same species (people, dogs, cats, cattle, or sheep) at the same camera within 0.5 h were regarded as independent photos [36]. Finally, we obtained 167 independent records of people and 114 independent photos of livestock. The number of the independent data of Reeves’s Pheasant movement was the value of the $\sigma_{m}^{2}$ of that hour. The kernel density estimation method in the “overlap” package of the R software was used to analyze the relationship between the diurnal movement patterns of Reeves’s Pheasants and human presence, and then the coefficient of overlap was calculated. The coefficient value ranged from 0 to 1, and a value closer to 1 means more overlap between activity of Reeves’s Pheasants and humans [37]. One-way ANOVA was used to analyze the difference in the coefficient value between people and livestock.

### 2.4. Effects of Human-Modified Habitat on the Movement Intensity of Reeves’s Pheasants

A partial least squares path model (PLS-PM), a statistical method for studying complex multivariate relationships, can reveal the direct or indirect relationship between observed and latent variables, and did not require any distributional assumptions to be met [38,39]. To evaluate the relationship between human-modified habitat and the movement intensity of Reeves’s Pheasants, we performed PLS-PM. Although our purpose was to obtain the response of Reeves’s Pheasants to human-modified habitat, reproductive behavior [40], habitat conditions [41], and individual body characteristics [11,42] can also affect the movement of wild animals. Therefore, we defined two models to describe sexual divergence. Whether individuals reproduced was considered in the female model, and other factors were considered as follows:

1. Disturbance pressure: The mean distance of each individual was derived from four human-modified habitats covering forest paths, paved roads, residential areas, and farmland.
2. Habitat conditions: We calculated the forest coverage and two landscape pattern indexes (aggregation index and mean patch area, reflecting the degree of landscape fragmentation: the lower the value, the higher the habitat fragmentation) within the home range of each individual using ArcGIS 10.2 and Fragstats 4.2. Forest coverage represents the degree of forest that was destroyed: the higher the forest coverage, the more complete the forest. Forest coverage was expressed as a percentage, and the arcsine square root transformation was carried out before analysis. We used two landscape pattern indices to conduct principal component analyses (PCAs) for males and females. PCAs can derive a few principal components from the original variables through linear transformation, so that they can retain as much information of the original variables as possible. Principal component analysis 1 (PCA1) was usually selected [43,44,45], and the larger the % variance, the better the PCA1 can explain the original variable. In this study, PCA1 explained 82.928% of the variance of males and 87.280% of that of females (Table A2). The two indices have equal weight in PCA1 of males and females, and were positively correlated: the higher the PCA1, the lower the degree of landscape fragmentation of individual habitat.
3. Body characteristics: A single morphological index was not sufficiently comprehensive to reflect the individual body size information. Based on the analysis methods of previous studies [43], we adopted four morphological indexes (wingspan length, tail feather length, body weight, and body length) to conduct PCAs for males and females. PCA1 explained 63.744% of the variance of males and explained 41.880% of the variance of females (Table A3). Body length has the highest weight in PCA1 of males and females. Many morphological studies assume that a positive correlation between morphological index measurements in PCA represents a positive correlation of body size [44]. Four morphological indexes of females and males were positively correlated: the higher the PCA1, the larger the body size of an individual. In addition, we calculated the ratio of body weight to tarsus length as a body condition index (BCI) [45]: the higher the BCI value, the better the individual body condition [44]. We used the variance inflation factor (VIF) to reduce the collinearity between variables. All variables were reserved in male and female models (VIF < 10).

In the PLS-PM, disturbance pressure, habitat conditions, body characteristics, movement intensity, and reproductive behavior were the manifest variables. To test the relationship between manifest variables and measurable variables, we deleted the measurable variables for which the loading was less than 0.7 [46]. We then used Dillon–Goldstein’s rho, which is considered better than Cronbach’s alpha, to evaluate whether these variables were unidimensional [38]. When each Dillon–Goldstein’s rho was larger than 0.7, indicators for manifest variables were unidimensional [47]. To evaluate the predictive ability of the model, we used the goodness-of-fit (GoF) index that describes the average prediction for the entire model. Finally, we deleted the body size, distance to residential area, and paved road in the male and female model, and distance to farmland in the male model. The GoF values of the two models were 58.56% (male) and 50.55% (female). The significance of the paths was tested by 999 bootstrap replicates. These results showed that the two PLS-PMs had better predictive ability [38,39,48].

## 3. Results

### 3.1. Movement Characteristics

**Figure 1:**
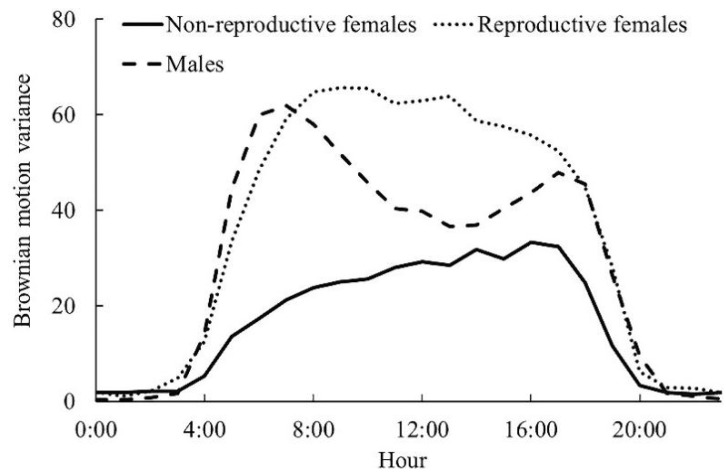
Diel movement patterns of Reeves’s Pheasants during the breeding season.

During the breeding season, males had two clear diurnal movement peaks at 6:00–8:00 am and 16:00–18:00 pm (Figure 1). Non-reproductive females (NRF) and reproductive females (RF) did not have such clear movement peaks. The movement intensity of RF from 8:00 to 16:00 was higher than that of males. There was a significant difference in movement intensity among different categories of Reeves’s Pheasant during the breeding season (F = 4.125, df = 2, *p* = 0.023). RF and males had a significantly higher movement intensity than NRF (*p* = 0.017).

### 3.2. Responses of Movement Patterns to Human Presence

**Figure 2:**
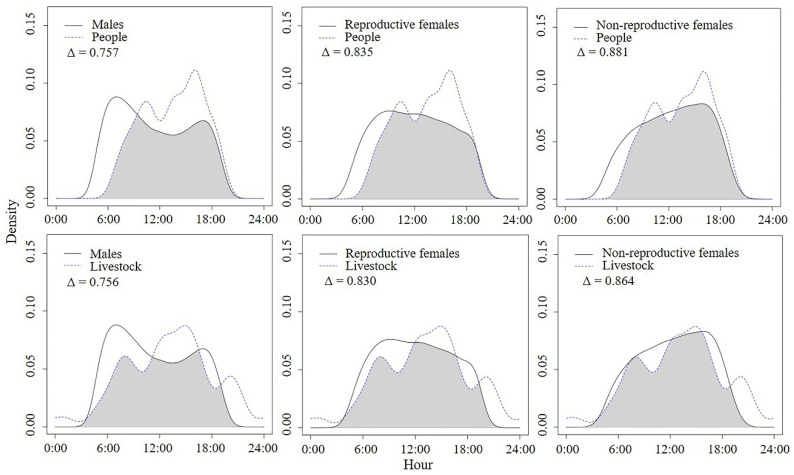
Relationships between diel movement patterns of Reeves’s Pheasants and human presence during the breeding season.

We observed a high degree of overlap between the diel movement patterns of Reeves’s Pheasants and humans (people and livestock) (Figure 2), and there was no significant difference in overlap degree between two types of human presence (F = 0.025, df =1, *p* = 0.881). We also observed a clear distance between the diurnal movement peaks of males and people. Males had the smallest overlap with people (0.757), and NRF had the largest overlap with people (0.880) and livestock (0.864).

### 3.3. Effects of Human-Modified Habitat on Movement Intensity

**Figure 3:**
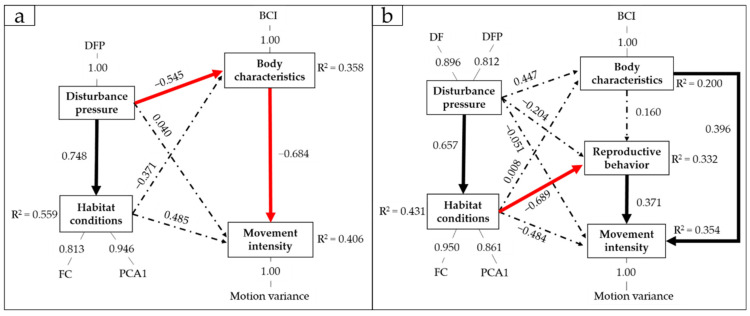
The path coefficients of PLS-PM prediction in (**a**) males and (**b**) females. DFP: distance to forest path; DF: distance to farmland; FC: forest coverage. The black solid lines indicate a significant positive correlation between variables. Red solid lines indicate a negative correlation. The dotted lines indicate that there is no significant correlation between variables.

PLS-PM showed that the correlation between human-modified habitat and the movement intensity of the Reeves’s Pheasant differed between sexes (Figure 3). We did not find a direct correlation between human-modified habitat and movement intensity.

For males, the disturbance pressure caused by human-modified habitats (forest paths) and habitat conditions had no significant direct correlation with movement intensity (Figure 3a). The distance to forest paths had a strong negative correlation with the body condition of male birds. The greater the distance, the poorer the body condition. Their body condition also had a negative correlation with movement intensity. The poorer the body condition, the higher the movement intensity. Thus, through affecting body condition, the distance to forest paths had a positive correlation with the movement intensity of males. The greater the distance, the higher the movement intensity.

For females, disturbance pressure and habitat conditions also had no direct correlation with movement intensity (Figure 3b). The distance to forest paths and farmland had a strong positive correlation with habitat conditions. The greater the distance, the better the habitat conditions. Habitat conditions also had a negative correlation with the possibility of reproduction of female birds. The better the habitat conditions, the lower the possibility of female reproduction. In addition, the possibility of female reproduction had a positive correlation with movement intensity. The lower the possibility of female reproduction, the lower the movement intensity. Thus, through affecting habitat conditions and reproductive behaviors, the distance to forest paths and farmland had a negative correlation with the movement intensity of females. The greater the distance, the lower the movement intensity.

## 4. Discussion

Since terrestrial forest-dwelling birds are highly vigilant, it is difficult for researchers to directly observe their behavior and accurately identify their individuals in the wild. Satellite tracking has been recognized as a better method to study the behavior of wildlife [12], especially of birds [49]. In our study, for example, it was extremely difficult to catch Reeves’s Pheasants and charge the tracker using solar energy in forests. These uncertainties have prevented researchers from obtaining sufficient data to conduct statistical analyses. We acknowledge that the number of individuals was relatively small, but we still examined the movement patterns of the Reeves’s Pheasant under human disturbance, and the results can provide insight for the protection of this species and other related bird species in the future.

### 4.1. Movement

Male Reeves’s Pheasants displayed two clear diurnal movement peaks, which is consistent with previous findings [30,50]. Researchers have found that the courtship and mating behaviors of terrestrial birds occur intensively in the morning or evening [51,52]. During the breeding season, male Reeves’s Pheasants occupy territories and attract females to mate [21], and their movement peaks are related to mating in addition to foraging, as noted in previous studies [30,50].

The movement intensity of non-reproductive females was significantly lower than that of reproductive females. Previous studies have shown that reproduction can significantly affect the movement intensity of female individuals [40]. During the breeding season, female Reeves’s Pheasants visit multiple male territories to choose mates [21]. After mating, female and male birds move separately, and females nest alone [21]. Females quickly leave their nest site after laying, before hatching, and hide their nest site in this way [53]. Therefore, the movement intensity of reproductive females was significantly higher than that of non-reproductive females, which is possibly related to mate selection, laying behaviors, and the need to supplement the energy required for reproduction by foraging [54].

### 4.2. Human Presence

Males showed two daily movement peaks at 6:00–8:00 am and 16:00–18:00 pm, which is inconsistent with previous studies [30]. Movement peaks of male Reeves’s Pheasants were timed to avoid contact with human beings, which is consistent with existing findings [29], and similar to the behaviors of other wild animals under human disturbance [3]. Previous studies have pointed out that livestock can have a strong impact on the movement patterns of wild animals [6,7]. However, we did not find that males avoided contact with livestock obviously, which is consistent with previous findings in the region [29]. Particularly, males’ movement was only slightly different from the previous record (one hour earlier). We collected data over a four-month period using 69 cameras, and obtained 167 people and 114 livestock records during this period. The data collection rate was almost one record per day for livestock and a bit more for people. Therefore, the inconsistency may be explained by the fact that there were fewer people and livestock in our study area, and thus the effect of disturbance pressure on Reeves’s Pheasants was weakened. Species’ movement patterns are also affected by their natural enemies [29]. For example, Reilly et al. [55] found that the movement of the Striped Skunk (*Mephitis mephitis*) was affected by its natural enemy the Coyote (*Canis latrans*). The natural enemies of Reeves’s Pheasants are raptors such as Black Kites (*Milvus migrans*), and small carnivores [50,56]. The daily movement peaks of Black Kites appear at 10:00–16:00 during the breeding season [57]. Therefore, the movement peaks of male Reeves’s Pheasants may also be affected by the presence of natural enemies.

We found that the effects of human presence on Reeves’s Pheasants differed between sexes. Due to the larger body and brighter feather, males are more likely to be discovered than females [22], and this may be the reason why males stayed further away. In addition, a previous study has pointed out that for sexually dimorphic birds, male individuals are more vulnerable to disturbance, because males need to spend more time foraging to recover costs [58]. Non-reproductive females had the highest degree of overlap with human presence, probably because non-reproductive females do not have to protect the nest site and young [22].

### 4.3. Human-Modified Habitat

Previous studies have pointed out that the impact of human disturbance on wildlife behavior is often manifested in the changes of habitat conditions that affect the animals’ reproduction and health, thus further affecting the animals’ behavior [59]. In this study, human-modified habitat had a significant correlation with the body characteristics of male Reeves’s Pheasants. The main goal of males is to obtain the opportunity to mate with females in the breeding season. The low density of undergrowth that we describe above allows males to show off their body characteristics by displaying the long tail feathers and vigorous posture [60]. For example, the Black-billed Capercaillie (*Tetrao parvirostris*) tends to choose open forest space as its courtship field [61]. Therefore, male individuals with better body condition may have higher possibilities to occupy the area close to forest paths as their own territory. The individuals with poor body condition may be displaced by individuals with strong combat effectiveness, leading to a high movement intensity. In addition, as a vigilant species, Reeves’s Pheasants usually choose to hide themselves when facing threats [22,30]. Therefore, the closer the distance to forest paths, the lower the movement intensity of males, which may be due to their courtship behaviors [61] or the fear of being preyed by natural enemies in open areas [56,60].

To choose a high-quality mate, females may prefer males with better body condition, while these males are likely to live near forest paths. For example, female Common Moorhens (*Gallinula chloropus*) prefer males with better body condition (higher BCI value) [62]. This may be the reason why female Reeves’s Pheasants located closer to human-modified habitats were more likely to reproduce. In addition, previous studies have pointed out that the probability of a forest being selected as the nest site by Reeves’s Pheasants is only 28.9% [21]. Reeves’s Pheasants build nests on the ground and have many nest predators [63,64]. Nesting near human-modified habitats such as roads and farmland [21,65] may reduce the risk of nest predation [59,66,67].

Under the conditions of rapid land use changes, it is assumed that some habitat characteristics attract wildlife, leading animals to choose poor-quality habitats instead of high-quality habitats, even though the poor-quality habitats provide an unsuitable environment for their reproduction or survival [68,69,70]. This hypothesis is usually known as the “ecological trap” [71]. During the study period, we found nine Reeves’s Pheasant nests in farmland, but none of the females hatched offspring successfully. The Reeves’s Pheasant is a species with highly sensitive alert behavior. The birds abandon their nests easily when encountering farmers, and farmers also take the birds’ nest eggs [21,64]. Therefore, Reeves’s Pheasants in this region may fall into an “ecological trap” made by human beings. At the same time, based on the ecological threshold principle [72], we believe that there should be a threshold value for whether the Reeves’s Pheasant will fall into an “ecological trap”. If the degree of human-modified habitat is lower than this threshold value, the Reeves’s Pheasant will fall into the “ecological trap”. If it is higher than this value, the Reeves’s Pheasant will avoid these habitats. For example, when the farmland area exceeds 25% of the total area, the Amur Tiger (*Panthera tigris altaica*) will not utilize this area [73]. At present, the degree of human-modified habitat in our study area may not have reached the threshold that would affect the survival of Reeves’s Pheasants. However, the range of this threshold value is unknown and needs further study.

## 5. Conclusions

We found that reproduction had a significant effect on the movement of females. Males shifted their movement peaks to earlier times in the day to avoid the presence peaks of humans. Human-modified habitat did not have a significant direct correlation with the movement intensity of Reeves’s Pheasants. Our findings were consistent with previous studies and showed that human-modified habitat had an indirect correlation with the movement of Reeves’s Pheasants through affecting male body characteristics and female reproductive behaviors. Furthermore, our study area was located near a village that was not a protected area, and we inferred that the Reeves’s Pheasant has formed a certain adaptive strategy under long-term disturbance. One caveat is that we only considered the distance to human-modified habitat in the PLS-PM, and it may not represent the total disturbance pressure caused by human-modified habitat.

We also highlighted the paucity of current research on terrestrial forest-dwelling birds regarding the factors affecting female reproduction or tolerance thresholds. In the context of vigorously carrying out ecological and environmental protection, we believe that future research should involve a long-term tracking and monitoring of this group of birds and promote the research work at deeper levels to provide a reference for the sustainable development of these species. At the same time, we suggest that the potential impact of different forms of human disturbance on wildlife should be considered in future protection planning.

## Data Availability

Raw data are available from the corresponding authors upon reasonable request.

## Appendix A

**Table A1:** Tracked information of Reeves’s Pheasants.

ID	Year	Date	Locations Quantity	Classification
x35	2020	26 March–24 May	1405	Male
x37	2020	22 March–16 April	584	Male
x43	2020	26 March–6 May	975	Male
x46	2020	31 March–1 June	1469	Male
x48	2020	28 March–14 May	997	Male
x50	2020	29 March–16 May	1142	Male
x51	2020	30 March–16 May	1108	Male
x52	2020	28 March–24 April	652	Male
x53	2020	31 March–16 May	1087	Male
x10	2021	30 March–7 May	913	Male
x12	2021	30 March–3 April	112	Male
x35	2021	13 May–31 May	431	Male
x51	2021	12 March–7 April	636	Male
x52	2021	5 June–20 June	353	Male
x60	2021	30 March–4 June	1562	Male
x61	2021	29 March–1 April; 4 April–11 May	937	Male
x85	2021	26 April–20 June	1315	Male
x87	2021	27 April–20 June	1282	Male
x91	2021	30 April–9 May	221	Male
c14	2020	26 March–16 May	1222	NRF
c14	2021	28 April–8 June	947	NRF
c16	2020	23 March–7 June	1616	NRF
c17	2020	27 March–1 April; 10 April–27 April; 19 May–1 June	885	RF
c21	2020	29 March–15 April; 4 May–24 May	908	RF
c25	2020	26 March–21 April; 1 May–28 May	1306	RF
c27	2020	26 March–18 April; 30 April–6 May	717	RF
c29	2020	23 March–22 April; 19 May–9 June	1245	RF
c32	2020	23 March–16 April	589	RF
c49	2020	29 March–5 May	896	RF
c55	2020	28 April–20 May	525	NRF
c56	2021	19 March–24 March; 22 April–27 April	255	NRF
c62	2021	30 March–17 April; 21 April–11 May; 27 May–18 June	1421	RF
c69	2021	14 March–7 April; 14 April–13 May; 27 May–31 May	1364	RF
c72	2021	15 March–24 March	234	NRF
c73	2021	17 March–23 April; 27 May–6 June	1126	RF
c74	2021	18 March–17 April; 25 May–15 June	1261	RF
c75	2021	19 March–23 April; 27 May–6 June	1072	RF
c78	2021	19 March–24 March; 22 April–11 May	587	NRF
c80	2021	20 March–7 April; 24 April–15 May	947	RF
c84	2021	23 April–16 May; 28 May–20 June	1091	RF
c86	2021	27 April–17 May; 22 May–20 June	1145	RF
c88	2021	26 April–16 May; 31 May–20 June	970	RF
c89	2021	27 April–17 May; 21 May–20 June	1212	RF
c92	2021	1 May–14 May	323	RF

RF: reproductive female; NRF: non-reproductive female.

**Table A2:** Principal component analysis of the landscape pattern index of 95%UD of male and female Reeves’s Pheasants.

Variables	Principal Component	
	Male PCA1	Female PCA1
Eigen value	1.659	1.746
%Variance	82.928	87.280
Aggregation index	0.911	0.934
Mean patch area	0.911	0.934

**Table A3:** Principal component analysis of morphologic characteristics of male and female Reeves’s Pheasants.

Variables	Principal Component		
	Male PCA1	Female PCA1	Female PCA2
Eigen value	2.550	1.675	1.062
%Variance	63.744	41.880	26.551
Body weight	0.672	0.439	0.651
Body length	0.939	0.783	−0.425
Wingspan length	0.620	0.391	0.634
Tail length	0.912	0.847	−0.237
